# Estimating a preference-based index for patients with myasthenia gravis from the MGQoL-6D measure

**DOI:** 10.1186/s12955-025-02370-2

**Published:** 2025-04-20

**Authors:** Richard Huan Xu, Richard Norman, Ji Liu, Dong Dong

**Affiliations:** 1 Department of Rehabilitation Sciences, Faculty of Health and Social Sciences, Hong Kong Polytechnic University, Hong Kong, China; 2 School of Population Health, Curtin University, Perth, Australia; 3 Beijing Aili Myasthenia Gravis Care Center, Beijing, China; 4 JC School of Public Health and Primary Care, Faculty of Medicine, The Chinese University of Hong Kong, Hong Kong, China

**Keywords:** Myasthenia Gravis, Preference-based measures, Valuation, Quality of life

## Abstract

**Objective:**

Myasthenia gravis is a chronic neuromuscular disease that causes weakness. It’s uncertain whether generic health instruments can adequately capture its impact. This study aimed to develop a scoring system to generate utility values for all health states defined by the myasthenia gravis quality of life 6-dimension (MGQoL-6D) classification in Chinese patients with myasthenia gravis (MG).

**Methods:**

The data used in this study were obtained from a web-based cross-sectional study conducted in China. Patients with MG were invited to complete an online discrete choice experiment (DCE) survey. Each participant completed 10 randomly assigned choice pairs from a set of 40 choice pairs, with each pair comprising two health states and a duration attribute. Conditional logistic regression analysis was employed to analyse the data, which included responses from 300 patients.

**Results:**

Utility decrements estimated by conditional logit regression were generally monotonic, with the largest decrements observed for emotion (− 0.419), social activity (− 0.323), and hobbies and fun activities (− 0.323). The MGQoL-6D utility scores ranged between − 0.559 and 1.

**Conclusion:**

This study established utility weights for the MGQoL-6D that can facilitate cost-utility analyses related to MG-related health interventions and technologies.

## Introduction

Myasthenia gravis (MG) is a chronic autoimmune neuromuscular disorder characterised by fluctuating muscle weakness and fatigue, which can significantly impair daily functioning and well-being [1]. This condition primarily affects voluntary muscles, leading to symptoms such as drooping eyelids, double vision, difficulty swallowing, and generalised muscle weakness. These symptoms can vary in severity, often worsening with activity, and can profoundly affect patients’ health-related quality of life (HRQoL) [2].

Myasthenia gravis quality of life 6-dimension (MGQoL-6D) is a newly developed MG-specific preference-based measure (PBM) that supports the estimation of quality-adjusted life years (QALYs) for the economic evaluation of MG. It was derived from the 15-item myasthenia gravis quality of life (MGQoL-15r) [3], a widely used MG-specific measure, through a comprehensive development progress in a large sample of Chinese patients with MG [4]. The descriptive system of MGQoL-6D has six dimensions: mobility, emotion, social activities, hobbies, and fun activities, work performance, and meeting family needs. A psychometric test confirmed the validity and robustness of the newly developed health status classification system (HCS) [4]. However, preference weights for MGQoL-6D are not currently available. To utilise the MGQoL-6D for economic evaluation, preference weights are essential. Thus, this study aimed to develop a scoring system that generates utility values for all health states, as defined by the classification system of the MGQoL-6D, using a 1 to 0 full health-dead scale to calculate QALYs.

The weighting of health states to reflect population preferences is widely used in health economics, in contrast with non-normative approaches such as summing across levels. Our data demonstrate that respondents do differentiate between shifts in health state depending on the dimension, and therefore, we prefer to reflect this in our scoring algorithm. An ongoing debate exists regarding whether to estimate the preferences of patients or the general population during the development of a value set for a PBM [5]. Both perspectives have valid arguments, and the choice between patient and general population preferences depends on the context and purpose of the value set. For health policy and resource allocation, general population preferences may be more relevant as they provide a broader societal perspective on the value of health states. However, for clinical decision-making and patient-centred care, patient preferences may be more appropriate as they directly reflect the experiences and priorities of those living with MG. Developing preference weights for the MGQoL-6D can bridge the gap between clinical assessment and patient experience, providing a robust tool to guide treatment choices, optimize resource use, and ensure that care for MG patients reflects their values and priorities. This is particularly vital in a condition like MG, where symptom fluctuation and treatment burden significantly affect daily life. Given that MG is a rare disease (RD), the general public may have limited knowledge about the condition, which could introduce bias if their preferences are used to estimate the value set. Therefore, we collected a patient sample and estimated preference weights within this population. This approach ensures that the value set accurately reflects the lived experiences and priorities of those directly affected by MG, thereby enhancing the relevance and applicability of the findings to patient-centred care and clinical decision-making.

## Methods

### Study design and participants

The data used in this study were obtained from a web-based cross-sectional study conducted in China between March and June 2024. All participants were recruited from the ‘*Beijing Aili Myasthenia Gravis Care Centre*’. This is one of the largest MG associations in China. Interested members were contacted and requested to participate in a specific online ‘survey group’. The researchers informed the participants about the study’s objectives and provided guidelines for completing the online questionnaire. The study protocol was approved by the Human Research Ethics Committee of the Hong Kong Polytechnic University (ref. no.: HSEARS20240226001), and informed consent was obtained from all participants.

### Measures

#### MGQoL-6D

The MGQoL-6D descriptive system was derived from MGQoL-15r. It comprises six items: social activities, hobbies and fun activities, work performance, meeting family needs, mobility, and emotion. Each item can be answered using a three-level scale ranging from 1 (no problem) to 3 (unable/extreme problem). Consequently, the MGQoL-6D descriptive system represents 3^6^ possible health statuses. It has demonstrated good psychometric properties in the Chinese MG population, as evaluated using both the classical test theory and item response theory methods [4].

### Profile selection for the discrete choice experiment (DCE) survey

The DCE combined with Time Trade-Off (DCE-TTO) method is a widely used approach to estimate preference weights for preference-based measures [6–9], like the MGQoL-6D because it effectively captures how individuals value different health states by integrating both choice-based and time-based valuation techniques. In a DCE, respondents are presented with hypothetical scenarios comparing pairs of health states defined by specific attributes, and they choose their preferred option, revealing relative preferences indirectly. The TTO component complements this by asking respondents to indicate how many years of life they would trade off to avoid a particular health state, providing a direct measure of utility anchored to a 0–1 scale (where 0 is equivalent to death and 1 is full health). This hybrid DCE-TTO method balances the cognitive simplicity and statistical efficiency of DCE with the precision of TTO in eliciting health state utilities.

The MGQoL-6D comprises six items with three response levels. We included a duration attribute to allow estimation of the trade-off between life expectancy and quality of life, which is intrinsic to the estimation of QALYs. When combined with the duration item (5, 10, 15, and 20 years), which was selected to be plausible for most respondents with sufficient spread to ensure discrimination between them, it resulted in over 2900 caring profiles (3^6^ × 4 = 2916). The durations were selected based on a literature review and consultations with patient associations and clinical experts, balancing the need to reflect realistic life expectancy impacts for MG patients in China with survey manageability. MG, a chronic condition with fluctuating severity, imposes long-term HRQoL challenges despite modern treatments preserving near-normal lifespan. These 5-year increments align with standard health state valuation practices (e.g., QLU-C10D), offering plausible horizons for respondents to assess health state trade-offs while supporting statistical modelling. Considering it is not feasible to include all possible combinations of profiles in a valuation survey, we used the D-optimality criterion, as recommended by International Society for Pharmacoeconomics and Outcomes Research, to determine the optimal experimental design for this study. This criterion aims to maximise the determinants of the information matrix for parameter estimation. Each item’s response level was treated as a categorical variable, whereas the duration item was treated as a continuous variable.

**Fig. 1:**
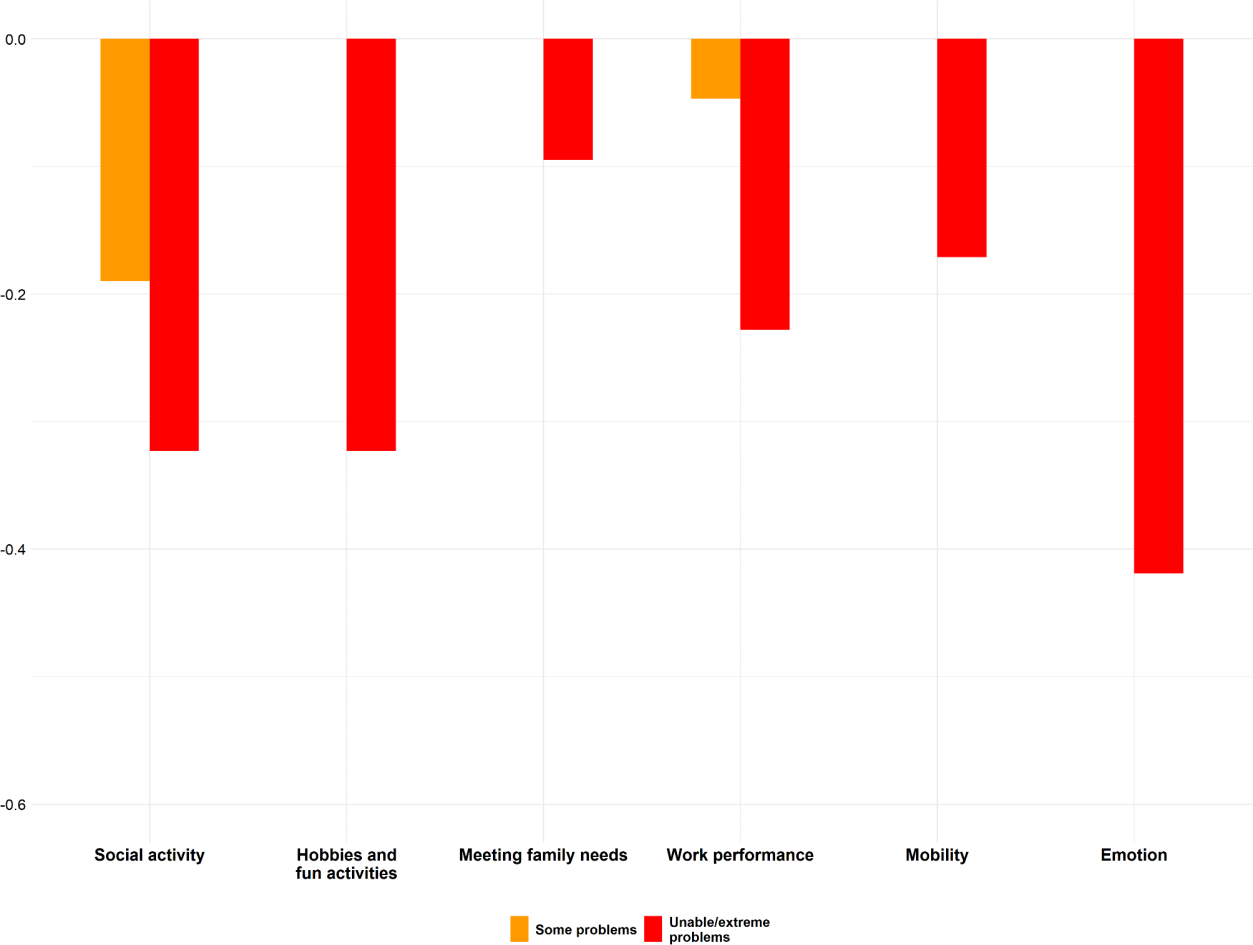
Utility decrements for the MGQoL-6D

A designed experiment was used to select 40 choice sets within four blocks to maximise statistical efficiency for estimating the main effects from the DCE data to estimate the MQGoL-6D utility weights. In the valuation survey, each participant was presented with 10 choice sets and asked to indicate their preference between two different MGQoL-6D health states (Situations A and B). Each was described by the 10 MGQoL-6D domains, with an additional attribute of survival duration. To reduce the cognitive burden of the choice task, each choice set included only four differing attributes (four of the six HRQoL attributes and duration), which were highlighted in yellow. The remaining three unhighlighted attributes were held constant, as illustrated in Fig. 1. The levels of the changing attributes were selected using a balanced incomplete block design, and a generator-based approach was used to determine how these four dimensions differed between options A and B. The levels of the attributes that were constant were selected using an orthogonal main-effects plan. The order in which participants observed the MGQoL-6D attributes in the DCE survey was randomised across participants; however, the order was maintained for each participant when completing all 10 choice sets. A pilot test was conducted with 10 patients with MG to confirm the feasibility and acceptability of this design before the formal valuation survey.

### DCE survey

The survey began with an information sheet, and informed consent was obtained before commencing the study. The survey consisted of five stages: First, respondents provided information about their socio-demographics, health, and HRQoL (EQ-5D-5 L). They also completed the MGQoL-6D classification system to familiarise themselves with the health state profiles used in the survey. Second, participants watched a video that explained DCE, its purpose, and how to answer its questions. Third, respondents completed a DCE question that included a dominated choice set, where Option A described a clearly better health status than Option B. Only data from those who answered this question correctly were included in the final analysis. Fourth, participants answered one block of 10 DCE questions. Finally, respondents answered four questions about the difficulty of understanding and completing the DCE tasks.

### Data analysis

A descriptive analysis was used to describe the participants’ sociodemographic characteristics and HRQoL. DCE data were analysed using a conditional fixed-effects logit model (CLM). To calculate the preference weights, the utility of option *j* (health status A or B) in choice set *s* for respondent *i* is described by the following formula:

*U*_*isj*_ *= αTIME*_*isj*_ *+ βX*_*isj*'_*TIME*_*isj*_ *+ ε*_*isj*_,

*i* = 1,…, *I respondents; j* = options A and B; *s* = 1,…, 40 choice sets.

where TIME_isj_ represents the survival time in option *j*, and the coefficient *α* denotes the utility associated with a life year. X′_isj_ is a vector of dummy variables representing the levels of MGQOL-6D health status described in each health state option *j* in a choice set, with *β* being the corresponding vector of coefficients associated with each level in each dimension within X′_isj_ for each life year. The error term ε_isj_ is assumed to follow a Gumbel distribution. Preference weights on the full health-dead 1 to 0 scale were generated using the marginal rate of substitution. The coefficient for each severity level of each item was divided by the duration coefficient (the ratio of β to α), which reflects the trade-offs between quality and quantity of life. Health-state utilities were generated by summing 1 and the relevant (negative) utility weights. All data analyses were conducted using STATA version 15 (StataCorp LP, Texas, USA).

## Results

### Feedback on the survey

Feedback on the DCE questionnaire was summarised. We found that 30% of participants indicated that the survey was more difficult than most other surveys they had completed (*n* = 89). Approximately 37 participants (12%) reported that the presentation of health status was unclear, and approximately 6% (*n* = 19) found it highly unclear. Approximately one-third (33%) of the participants found it difficult or highly difficult to choose between pairs of health statuses.

### Demographics and HRQoL of participants

Table 1 presents the participants’ sociodemographic characteristics. Of the 300 patients, 53.3% were female, 59% were urban residents, and 24.9% had completed their tertiary education. Regarding the types of MG, 31% were ocular plus generalised, and 24.3% were ocular plus bulbar and generalised. The mean age of the participants was 46 years (SD = 12.3).

**Table 1:** Patients’ background characteristics

	*n*	%
Sex		
Female	160	53.3
Male	140	46.7
Family registry		
Urban	177	59
Rural	123	41
Education		
Primary school	20	6.7
Secondary school	205	68.4
Tertiary school	75	24.9
Marital status		
Single	52	17.3
Married	206	68.7
Divorced/Widow(er)	42	14.0
MG types		
Generalized	33	11.0
Bulbar	1	0.3
Ocular	46	15.3
Bulbar + Generalized	38	12.7
Ocular + Generalized	93	31.0
Ocular + Bulbar	13	4.3
Ocular + Bulbar + Generalized	73	24.3
	Mean (range)	Standard deviation
Age	46 (18–78)	12.3

Table 2 presents the participants’ HRQoL according to the EQ-5D-5 L and MGQoL-6D. Regarding the MGQoL-6D, 54.3% and 51.7% of participants reported no problems with mobility and emotion, respectively, which is higher than the corresponding domains of mobility (31%) and anxiety/depression (49.3%) of the EQ-5D-5 L. A higher proportion of patients reported extreme problems in four out of five domains of the EQ-5D-5 L (ranging from 39 to 77.7%) compared to the domains of the MGQoL-6D (ranging from 10.4 to 28.3%). The mean EQ VAS score was 72.4 (SD = 19.4).

**Table 2:** Response on MGQoL-6D and EQ-5D-5 L

Measures	Response (%)					Mean (SD)
	Level 1	Level 2	Level 3	Level 4	Level 5	
MGQoL-6D				-	-	-
Social activity	42	38	20	-	-	-
Hobbies and fun activities	38.7	42.3	19	-	-	-
Meeting family needs	37	44.7	18.3	-	-	-
Work performance (including work at home)	31.4	40.3	28.3	-	-	-
Mobility	54.3	35.3	10.4	-	-	-
Emotion	51.7	34.7	13.6	-	-	-
EQ-5D-5 L						
Mobility	31	4.3	5.3	57.3	2	-
Self-care	1.0	16.7	3	1.7	77.7	-
Usual activities	1.7	27.7	2.7	3.7	64.3	-
Pain/discomfort	51.3	0.3	8.1	1.0	39.0	-
Anxiety/Depression	49.3	3.3	8.3	3.3	35.7	-
EQ VAS	-	-	-	-	-	72.4(19.4)

### Conditional logit utility decrements

The results of CLM analysis are presented in Table 3. The last column represents the preference weights (or utility decrements) assigned to each severity level of the MGQoL-6D items, expressed as a reduction in utility on a scale anchored at death = 0 and full health = 1. The calculation involves dividing the beta coefficient (β) for each severity level by the time coefficient (α) and anchoring the results to ensure consistency with the 0–1 utility scale. For example, for Social Activity at severity level 3, the β is -0.034, and the utility decrement is calculated as -0.034 / 0.105 ≈ -0.323, meaning this health state reduces utility by 0.323 relative to the baseline. Negative values signify a loss in quality of life, with larger negative numbers (e.g., -0.419 for Emotion at level 3) indicating greater perceived impact. Parameters with non-significant β values suggest minimal or no meaningful utility change, while significant decrements reflect stronger patient preferences for avoiding those health states. A total of 7/13 parameters showed significant coefficients. The largest utility decrements were observed for the domain of emotion, with a decrement of − 0.419 for Level 3 (unable/extreme problems). The domain with the second-largest utility decrement was social activity and hobbies and fun activities. Utility decrements for Level 3 (unable/extreme) were statistically significant for five domains, but only for one Level 2 (social activity).

**Table 3:** Conditional logit utility decrements

Parameter	Severity level	Beta (β)	SE	Anchored Consistent model Utility decrement
Time coefficient (α)	Linear	0.105^***^	0.013	
Social activity	2	-0.02^**^	0.007	-0.19
	3	-0.034^***^	0.007	-0.323
Hobbies and fun activities	2	0.001	0.005	0.009
	3	-0.034^***^	0.006	-0.323
Meeting family needs	2	0.02	0.006	0.19
	3	-0.01	0.007	-0.095
Work performance (including work at home)	2	-0.005	0.006	-0.047
	3	-0.024^**^	0.007	-0.228
Mobility	2	0.001	0.006	0.009
	3	-0.018^***^	0.008	-0.171
Emotion	2	0.009	0.007	0.086
	3	-0.044^***^	0.008	-0.419

Level 2 = some problems; Level 3 = unable/extreme problems;

SE = Standard error

** *p* < 0.01

*** *p* < 0.001

### Estimation of the MGQoL-6D utility scores

The utility scores for the MGQoL-6D value set were calculated using the weights generated by CLM. A value of 1, which indicates full health, is assigned to individuals who self-report ‘no problem’ for all six MGQoL-6D items that contribute to the scoring, meaning they are assigned Level 1 for all six attributes of the MGQoL-6D. The utility score for other health states can be calculated as follows: one minus the sum of the utility decrements for the selected levels of each attribute. The formula for the utility score (*U*) is:

$$\:U=1-{\sum\:}_{i=1}^{6}\varDelta\:{U}_{i}\:{}^{}$$

Where ΔU_i_​ is the utility decrement for the severity level of the i-th parameter (i.e., Social Activity, Hobbies and Fun Activities, Meeting Family Needs, Work Performance, Mobility, and Emotion), as derived from the anchored consistent model (see Table 3). For example, if a patient’s health state is classified as ‘231111’ using the descriptive system of MGQoL-6D (i.e., some problems with social activities, extreme problems with hobbies and fun activities, but no problems with meeting family needs, work performance, mobility, and emotion), the utility score for this health status would be estimated as: 1–0.19 − 0.323–0 − 0 − 0 − 0 = 0.487. The worst possible health state, known as the PITS state (333333), has a value of -0.559.

## Discussion

This paper describes the estimation of preference weights for the MGQoL-6D in a sample of 300 patients with MG in China. The MGQoL-6D descriptive system and its value set can be used to calculate QALYs and assess the cost-effectiveness of new and existing MG interventions. However, the preference weights estimated in this study were based on MG patients’ preferences rather than those of the general population, an approach adopted by most previous valuation studies, such as Duchenne muscular dystrophy quality of life (DMD-QoL) [8]. The impact of this methodological choice on the value-set estimation is unclear. Nonetheless, it provides a unique perspective on how patients with RDs value their health using a specific HRQoL instrument. Future research should estimate preference weights based on the general population to compare with those generated in this study, further contributing to the field of patient-reported outcome measures.

MG is a chronic autoimmune disorder that causes weakness in the skeletal muscles responsible for breathing and movement, including those in the arms and legs. We found that emotions significantly influence the HRQoL of patients with MG. This finding is noteworthy because, compared to the utility weights of DMD-QoL—another RD—estimated based on the general population’s preferences, the utility decrease for the ‘worried’ domain is smaller than that of many other physical health-related domains [8]. Previous studies have indicated that the unpredictable nature of RDs, including MG, with their fluctuating symptoms and the potential for sudden exacerbations, may lead to heightened anxiety and stress [10, 11]. Moreover, the lack of widespread awareness and understanding of MG can result in delayed diagnoses and misdiagnoses, further exacerbating emotional distress. Physical limitations imposed by the disease can also affect a patient’s self-esteem and sense of independence, leading to depression and diminished HRQoL. Given these findings, addressing the emotional well-being of patients with MG is crucial to their overall health. In the future, it would be valuable to compare the preference weights of the MGQoL-6D developed based on the general population with our results. This comparison can help us better understand which perspective should be used to support the economic evaluation of RDs.

Additionally, we found statistically insignificant utility decrements for some levels, especially, at Level 2, across five MGQoL-6D domains. Several studies have reported this similar issue [8, 12, 13]. However, these nonsignificant results for Level 2 severity in several domains do not indicate a major problem. They likely stem from the relatively small mean decrements (≤ 0.03) and preference heterogeneity among individuals. Moreover, examining the response distribution reveals that 35–45% of patients select Level 2 options. This suggests they may not perceive their health state as very severe, making them reluctant to trade off time to prevent it.

Although the MGQoL-6D was derived from the MGQoL-15r, which was designed to measure HRQoL in MG, some domains are common across different RDs. For instance, ‘meeting family needs’ is rarely assessed by generic PBMs but has been identified as important for patients with RD and their families in studies [14, 15]. Many patients with RD can work but must do so from home. Research indicates that work is crucial for patients with RDs, making them feel useful [16]. We included this item in the MGQoL-6D descriptive system to accurately address their specific needs. However, some domains in generic PBMs, including EQ-5D, such as ‘self-care’ and ‘usual activities’, are less relevant to patients with RD, as most cannot care for themselves or perform the usual activities described by EQ-5D. In this study, only 1% and 1.7% of participants reported no problems in these two domains. Therefore, the MGQoL-6D may have the potential for application not only in MG but also in other RD studies to facilitate economic evaluation.

Our findings indicate that a subset of participants found the DCE questionnaire challenging or struggled to interpret the health status presentations. However, the majority did not perceive the survey as overly difficult, with only 12% citing clarity issues and 33% reporting difficulty in making choices. This suggests that, despite some cognitive demands, the task was manageable for most respondents. To address these challenges in future studies, we could consider integrating interactive tutorials or streamlined visual aids to improve comprehension of health status descriptions. Additionally, conducting pre-tests through cognitive interviews or pilot phases could help pinpoint and resolve potential ambiguities. Such enhancements may minimize perceived difficulty for rare disease patients while preserving the methodological rigor essential for effective preference elicitation.

This study has several limitations. First, only 300 patients were involved in the survey. Although some valuation studies had similar or fewer participants, the number of observations per state in our study was below the recommended threshold. This may introduce uncertainties in the estimation of preference weights for the MGQoL-6D. Second, our sample lacked a sufficient number of young patients. With a mean age of approximately 46 years, the small percentage of younger participants may indicate inherent selection bias, potentially skewing the estimation of preference weights. Third, although MGQoL-6D’s psychometric properties have been tested in the MG population, its performance, including responsiveness, has not been confirmed in clinical practice. Because most domains of the MGQoL-6D focus on social well-being, its sensitivity in clinical settings remains unknown. This uncertainty may affect the practical application of the instrument. Finally, similar to other web-based valuation studies, our survey process may have introduced an information bias. Despite conducting a pilot study and including an introductory video for the DCE survey, respondents who were unfamiliar with online survey methods might have provided systematically inappropriate responses.

## Conclusion

This study provides the preliminary preference weights for the MGQoL-6D, a new condition-specific patient-reported outcome measure for MG based on MG patients’ preferences. The resulting scoring algorithm and value set can help clinicians and policymakers evaluate the impact of MG treatments on quality of life in cost-utility analyses. This facilitates evidence-based decisions regarding resource allocation.

## Data Availability

No datasets were generated or analysed during the current study.
